# Pulmonary Metastases From Primary Thigh Leiomyosarcoma: A Case Report and Review of the Literature

**DOI:** 10.7759/cureus.39562

**Published:** 2023-05-27

**Authors:** Prabasha Weeraddana, Hedaya Othman, Ragaa Elkabbani, Susanna Josey, Nisha Nepal, Eric Ma

**Affiliations:** 1 Internal medicine, Danbury Hospital, Danbury, USA; 2 Internal Medicine, Danbury Hospital, Danbury, USA; 3 Pathology, Danbury Hospital, Danbury, USA; 4 Hematology and Oncology, Danbury Hospital, Danbury, USA

**Keywords:** sst, tumor of smooth muscle cell, “literature review”, leiomyosarcoma of the thigh, pulmonary metastasis, leiomyosarcoma of somatic soft tissues

## Abstract

Leiomyosarcoma is a rare type of tumor of smooth muscle cells that can occur anywhere in the body. However, it typically occurs in the retroperitoneum, intra-abdominal sites, and uterus in people over 65. Here is a case of a 71-year-old male with a history of melanoma of the skin who presented with a rapidly enlarging, non-tender lump at his left lateral thigh area that was later diagnosed as pleomorphic dedifferentiated leiomyosarcoma. The patient underwent radical resection of the tumor and the attached vastus lateralis muscle and partial lateral collateral ligament, followed by radiation therapy to the resected site. He had no evidence of tumor recurrence on follow-up imaging for several months until he was found to have metastatic disease to the lungs on a surveillance CT one year later. A biopsy confirmed that the lung nodules were leiomyosarcoma metastases, and the patient was started on chemotherapy and stereotactic body radiation therapy (SBRT). Upon reviewing the literature, a few cases of leiomyosarcoma arising from the thigh muscles were found.

## Introduction

Leiomyosarcoma is a rare type of cancer that arises from the smooth muscle cells. It can develop in various parts of the body, including the uterus, gastrointestinal tract, and retroperitoneum. Only 5%-10% of all malignancies are soft tissue sarcomas, which make up 0.7% of all malignancies [1]. While there have been reported cases of leiomyosarcoma occurring in places such as the oral cavity, conjunctiva, and inferior vena cava, there are only a few documented instances of the tumor originating in the lower extremity muscles. These are categorized as leiomyosarcomas of the somatic soft tissues (SST). Leiomyosarcomas often metastasize to the lungs, liver, skin, bone, and mesenteric or omental soft tissues [2]. Routine chest X-rays and CT scans are recommended for detecting asymptomatic pulmonary metastases. Treatment for lung metastasis typically involves surgery, chemotherapy, and radiation therapy [3]. Due to the rare occurrence and unusual site of origin of leiomyosarcoma, this case is of particular interest. Here we present a case of a 71-year-old male who initially presented with swelling on his left thigh, later diagnosed as leiomyosarcoma. Following resection of the tumor and radiation therapy, he was free of disease but was diagnosed with lung metastases about a year later. 

## Case presentation

A 71-year-old male presented to the primary care physician's office complaining of a tender lump above his left lateral knee. He first noted the lump two months ago, and it was initially non-tender. He did not seek any medical evaluation initially. Over the subsequent two months, it rapidly enlarged to a size of 5 cm in diameter. Later, he developed mild discomfort over the lump but denied any redness, drainage, or open wounds. He had a past medical history of hyperlipidemia and melanoma of the skin, which was resected. He had no history of recent trauma or injury and denied fever, chills, weight loss, nausea, or loss of appetite. He had no limitations in mobility. He is a non-smoker and uses alcohol occasionally. His family medical history was notable for a brother with lung cancer and his daughter with colorectal carcinoma.

Examination revealed a left lateral thigh lump 4 x 5 cm in dimension with mild tenderness on palpation and an indurated area inferior to the center of the lump. It was firm in consistency with no redness, open wound, rash, or any other abnormal skin lesions in the leg. Both lower extremities were warm to the touch, and a positive distal pulse was palpated. His baseline blood workup showed white blood cells (WBC) 5.5 × 109/L, absolute neutrophil count 3440/mm3, hemoglobin (Hb) 15.4 g/dl, platelets 247000/ml, creatinine 1.10 mg/dl, calcium 9.6 mg/dl, albumin 4.4 g/dl, total protein 7.0 g/dl, aspartate transaminase (AST) 20 U/L, alanine transaminase (ALT) 15 U/L, alkaline phosphate (AKP) 81 U/L, lactate dehydrogenase (LDH) 182 U/L and uric acid 6.4 mg/dl.

**Figure 1:**
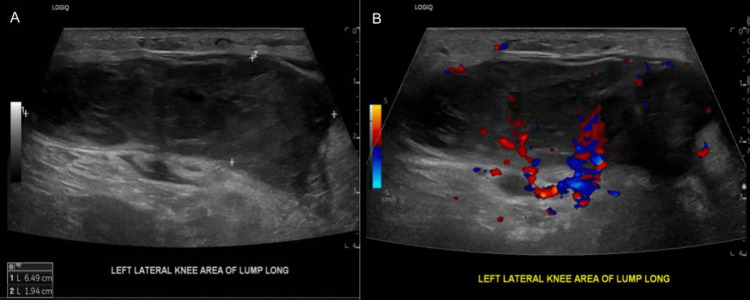
Ultrasound scan of the lump above left lateral knee area A) longitudinal view showed a heterogeneous hypoechoic mass, B) longitudinal view with Doppler study where the neovascular formation was visualized.

**Figure 2:**
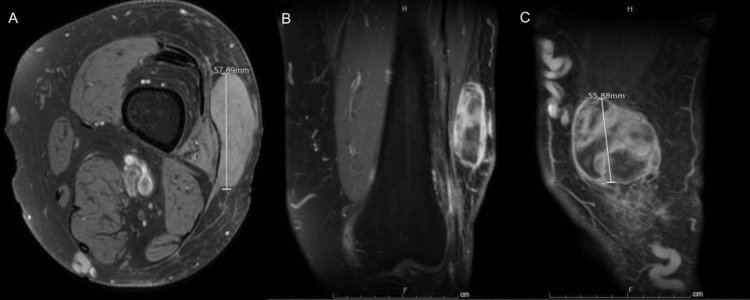
Magnetic resonance imaging (MRI) with and without contrast showed heterogeneous solid enhancing soft tissue mass A) - axial view B) - coronal view C) - sagittal view.

**Figure 3:**
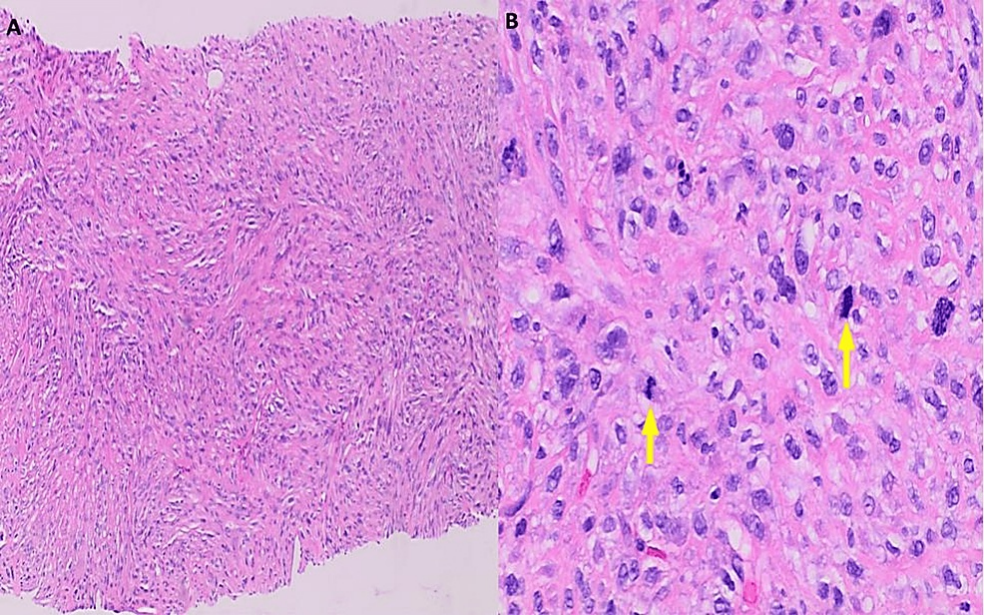
Ultrasound-guided core needle biopsy of left thigh mass displayed A) pleomorphic spindle cell sarcoma, high-grade with tumor cells arranged in storiform pattern (H&E, x100), B) on higher magnification sheets of polygonal, spindled, and epithelioid cells are seen, with eosinophilic cytoplasm, marked nuclear pleomorphic, multinucleation and conspicuous mitotic activity (yellow arrows), including atypical forms (H&E, x400). H&E: hematoxylin and eosin stain

**Figure 4:**
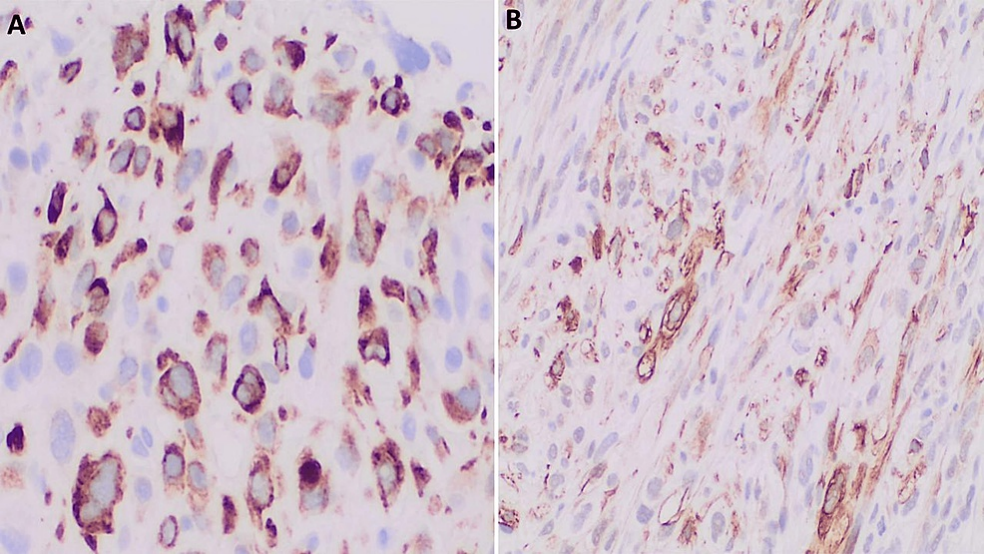
Immunohistochemical analysis showed tumor cells with multifocal positivity for A) Desmin (400x) and B) smooth muscle actin (SMA) (200x) in a subset of neoplastic cells, while caldesmon, SOX-10, and SATB2 were negative. Additionally, the melanoma markers (Melan A and HMB45) and epithelial (CK AE1:AE3) immunostains were negative.

Ultrasound of the left leg was performed, and it showed a solid vascular mass within the nodule measuring 6.5 x 1.9 x 5.5 cm with surrounding soft tissue edema (Figure 1). A Follow-up magnetic resonance imaging (MRI) with and without contrast was performed to further characterize the mass. MRI showed a heterogeneous, solid 5.8 cm mass in the anterolateral distal left thigh, which was predominantly subcutaneous, but also infiltrated a small portion of the musculotendinous junction of the vastus lateralis (Figure 2). The patient underwent an ultrasound-guided needle biopsy of the thigh mass. Histology revealed a high-grade pleomorphic spindle cell sarcoma, mostly suggestive of leiomyosarcoma (Figures 3-4). Subsequently, the patient underwent a CT chest, abdomen, and pelvis with contrast to rule out metastatic disease for which there was no evidence.

**Figure 5:**
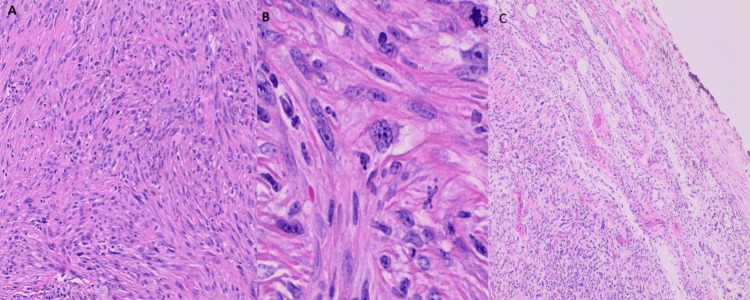
Histology of left thigh mass, resection showed (A-H&E 100x) fascicular architecture of high-grade pleomorphic spindle cell sarcoma, most suggestive of leiomyosarcoma, composed of cellular, intersecting fascicles of spindle cells, focally invading skeletal muscle, B-H&E 400x - pleomorphic cells have abundant eosinophilic, fibrillary cytoplasm, multinucleated giant cells, and mitotic figures can be seen.​ C - H&E 40x - tumor cells present on ink at the anterior surgical margin.

**Figure 6:**
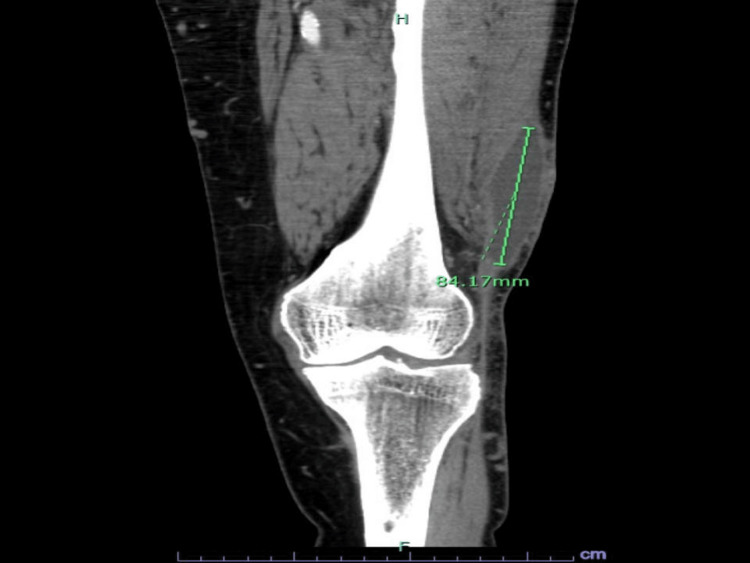
Left knee CT with contrast showed a postoperative seroma along the distal and lateral aspect of the left thigh superficial to the vastus lateralis musculature.

Afterward, the patient underwent radical resection of the left thigh mass with the parts of the vastus lateralis muscle and lateral collateral ligament attached to it. The pathology showed a grade 3 pleomorphic dedifferentiated leiomyosarcoma measuring 7.6 cm with the focal invasion of skeletal muscle with a positive anterior margin for malignant cells but negative margins otherwise (Figure 5). He received radiation therapy to the left knee after the surgical resection. He was followed up with a left knee CT with contrast one-month status post radiation completion. The CT showed a postoperative seroma along the distal and lateral aspect of the left thigh superficial to the vastus lateralis musculature measuring 2.6 cm x 5.1 cm x 8.4 cm (Figure 6). There was no apparent evidence of a recurrent or residual tumor. CT chest, abdomen/pelvis three months and six months following radiation therapy did not display disease recurrence either. Follow-up MRIs of the left knee/distal thigh without and with contrast at six and twelve months were also negative for tumor recurrence. 

**Figure 7:**
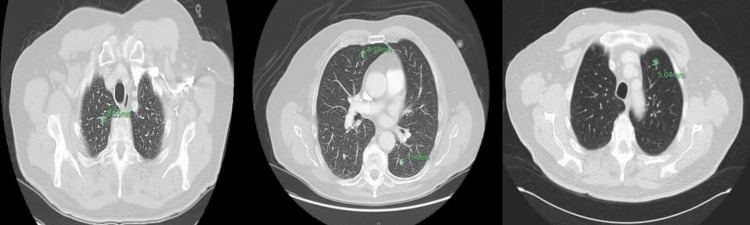
CT chest axial view one year after radiation therapy displayed the presence of six new pulmonary nodules.

**Figure 8:**
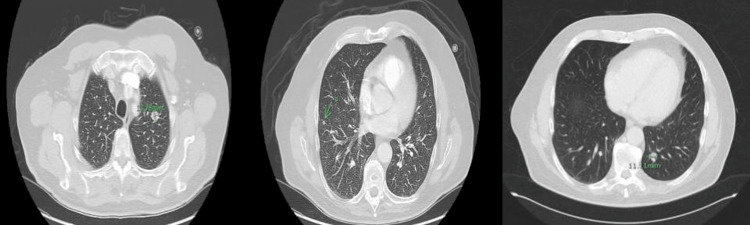
CT chest axial view one year after radiation therapy displayed the presence of six new pulmonary nodules (rest of nodules)

**Figure 9:**
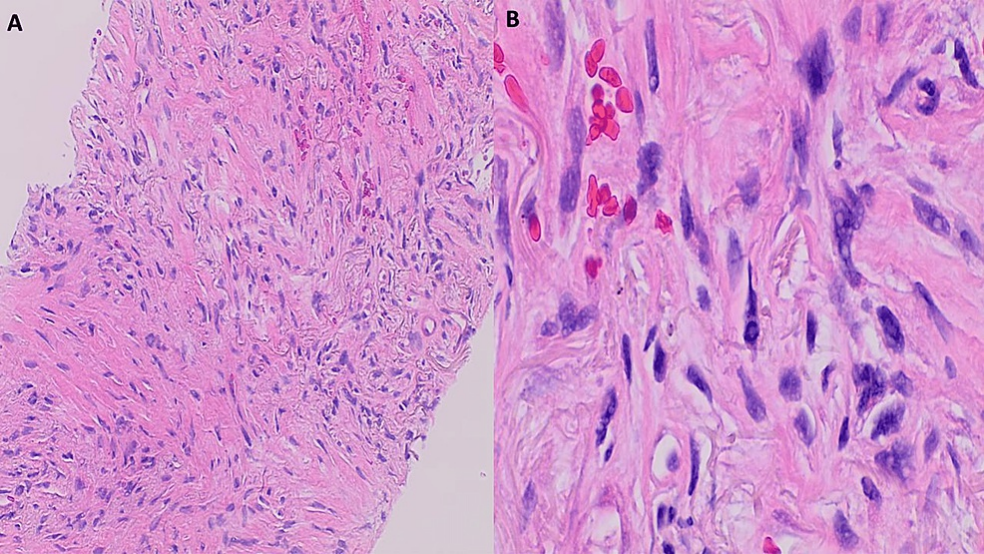
CT-guided needle biopsy of the left lung, upper lobe, showed A) lung parenchymal infiltration by a spindle cell malignant neoplasm with pleomorphic cells that are morphologically similar to the patient's prior known diagnosis of pleomorphic/dedifferentiated leiomyosarcoma, FNCLCC grade 3, (100X, H&E)​. B) tumor cells with bizarre nuclei and surrounding stromal hyalinization (400X, H&E).

Unfortunately, follow-up surveillance with CT chest, abdomen, and pelvis one year after radiation therapy displayed the presence of six new pulmonary nodules, which were highly suspicious for metastatic disease (Figures 7-8). The patient underwent a CT-guided biopsy of the left upper lung nodule, which was consistent with the histology of the leiomyosarcoma from the thigh (Figure 9).

**Figure 10:**
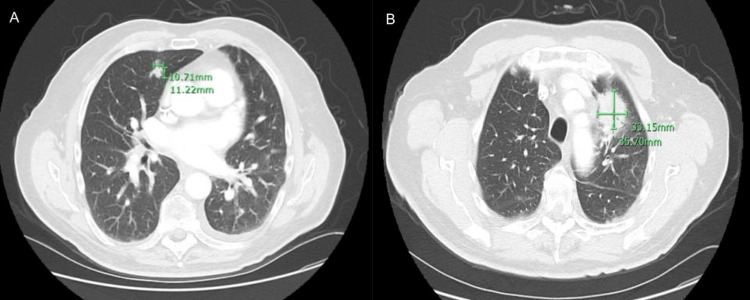
CT chest with contrast showed progression of the metastatic disease with an interval increase in the size of a right middle lobe nodule measuring 10.71 mm (A - axial view) and an interval increase in the size of a left upper lobe nodule at 33.15 x 35.70 mm (B - axial view).

Because of this new evidence of metastasis and, consequently, new stage 4 status, the patient started chemotherapy with adriamycin and stereotactic body radiation therapy (SBRT) to the left upper lobe of the lung. Follow-up CT chest with contrast showed progression of the metastatic disease with an interval increase in the size of a left upper lobe nodule at 3.3 x 3.6 cm and an interval increase in the size of a right middle lobe nodule measuring 1.1 cm in size (Figure 10). Because of the increase in the size of the nodules, the patient began a new chemotherapy regimen with liposomal doxorubicin 30 mg/m2 IV every four weeks.

## Discussion

A malignant smooth muscle soft tissue sarcoma called leiomyosarcoma may develop anywhere in the body. Leiomyosarcomas are uncommon, making up just 10% of all soft tissue sarcomas [1]. They do not vary by gender and often develop in people over the age of 65. Intra-abdominal sites (35%), the uterus (30%), the extremities (19%), the head and neck, as well as the chest (16%) are the sites of origin that have been documented [4]. Leiomyosarcoma of SSTs describes tumors of smooth muscle origin that occur outside the typical sites, such as in the extremities. It makes up a very minor portion of all soft tissue sarcomas. Due to regional and demographic variations, it is difficult to pinpoint the precise incidence of leiomyosarcoma of the thigh or leg [5]. As they are exceedingly low in occurrence, leiomyosarcoma of SST cases are not widely investigated and need more documentation. One study found that the majority of SST leiomyosarcomas are of vascular origin, and large tumors of this sort are more likely to be disrupted and metastasize [6].

The exact cause of leiomyosarcoma is not fully understood, but certain risk factors have been identified that may increase the likelihood of developing this type of cancer. Inherited genetic conditions such as Li-Fraumeni syndrome, neurofibromatosis type 1, hereditary retinoblastoma, exposure to radiation or chemicals, and older age may increase the risk of developing leiomyosarcoma [5]. However, having one or more of these risk factors does not necessarily mean that a person will develop leiomyosarcoma. Many people with leiomyosarcoma do not have any known risk factors, as with our patient. Although the symptoms differ depending on the location and stage of the malignancy, typical symptoms include fatigue, fever, weight loss, malaise, nausea, and vomiting. While pain is usually absent or if present, very mild, a palpable mass or notable swelling is often apparent, as in this case, because of the tumor’s rapid development [7]. Large tumors can impact patients' mobility, particularly if they arise in the lower extremities, but in this case, the patient’s mobility was not affected.

A primary diagnosis of the tumor can be established using simple imaging methods such as ultrasonography, CT scanning, or MRI [8]. After reviewing the histology, a definitive diagnosis can be made. Under a microscope, one can observe how spindle cells proliferate to produce rough bundles and fascicles. The typical cigar-appearance of the cells’ nuclei is also discernible, in addition to cytologic atypia and mitotic figures [2].

Leiomyosarcoma of the limb region can present with symptoms similar to other conditions, making it essential to consider it as a differential diagnosis and to rule out other potential causes. Liposarcoma, synovial sarcoma, angiosarcoma, neurofibroma, and fibrosarcoma are some of the conditions that can be considered in the differential diagnosis of leiomyosarcoma of the extremities [9]. It is essential to distinguish between these conditions because they may require different treatments and also have different prognoses.

Leiomyosarcoma can metastasize or spread to other parts of the body through the bloodstream or lymphatic system. The most common sites of metastasis for leiomyosarcoma are the lungs, liver, and bone. Metastasis to other soft tissues and organs, such as the brain or skin, is less common. It is estimated that up to 40%-50% of individuals with leiomyosarcoma develop pulmonary metastases [10]. In our case, the patient developed pulmonary metastasis after approximately one year. A patient with SST leiomyosarcomas above the age of 62, has a tumor larger than 4 cm, necrosis, grade of the French Federation Nationale des Centers de Lutte Contre le Cancer (FNCLCC), has vascular invasion, or has undergone prior intralesional surgery has a greater risk of metastasis [11]. Our patient had several of these factors increasing his risk for metastasis, including the age of diagnosis (71 years), tumor size of about 7.6 cm, intralesional disruption via ultrasound-guided biopsy before complete surgical resection, and an FFCC grade 3 tumor due to a positive excisional margin. Despite receiving radiation therapy after surgery to reduce the risk of metastasis, he ultimately developed metastasis one year after completing his therapy.

In general, leiomyosarcoma is an aggressive cancer that tends to grow and spread quickly. The five-year survival rate for individuals with leiomyosarcoma ranges from 50% to 70%, depending on the cancer stage in addition to other factors [12]. Factors that can negatively affect the prognosis of leiomyosarcoma include the presence of metastasis, tumor size, tumor grade, and tumor location. Tumors that are larger, high grade, and located in areas that are difficult to treat may be more challenging to manage and may have a poorer prognosis [13]. Mankin et al. found no significant effect on survival outcomes based on gender, age, site, use of adjuvant therapy, or local tumor recurrence [14].

Several case reports and studies have reported on leiomyosarcoma of the thigh [6,13-16]. T. Rizwan et al. described a case of a 65-year-old male patient with poorly differentiated leiomyosarcoma involving the muscles of the posterior compartment of the left thigh, with no metastasis to the liver, lung, or bone [6]. A literature search conducted in Japan found that the lung was the first metastasis site in 53 cases of leiomyosarcoma, with the primary lesion located in various sites such as the uterus, urinary bladder, thigh, and inferior vena cava. The mean interval from diagnosis of the primary tumor to detection of the pulmonary metastasis was 31 months. In some cases, the metastasis was detected over five years after diagnosis of the primary tumor [15]. Mizoshiri et al. also reported two cases of leiomyosarcoma of an extremity with hepatic metastasis, and one of those originated from the left thigh. The hepatic lesions were diagnosed three years after surgical resection of the primary tumor [13]. The summary of previously documented cases of leiomyosarcoma having primary origin from the thigh is shown in Table 1.

**Table 1:** Case reports/studies to date of leiomyosarcoma having primary origin from the thigh.

Case	Authors	Age/Gender	Primary Tumor Location	Metastatic Sites	Diagnosis
1	Rizwan et al. [6]	65-year-old male	Posterior compartment of left thigh muscles	No metastasis was detected in the liver, lung, or bone.	Leiomyosarcoma
2	Itoga et al. [15]	53 cases, median age 58 years old, female to male ratio approximately 3 to 1	Uterus, urinary bladder, thigh, inferior vena cava	Lung	Leiomyosarcoma
3	Mizoshiri et al. [13]	60-year-old Japanese male	Left thigh	Liver	Metastasis of leiomyosarcoma
4	Mankin et al. [14]	19 cases, median age 56 ± 19 years, no significant difference in the male-to-female ratio	Thigh	Lung, liver, viscera, bone, distant muscle	Metastasis of leiomyosarcoma
5	Lee et al. [16]	66-year-old male	Left thigh	No metastasis.	Leiomyosarcoma

Surgical resection followed by radiation therapy is the primary treatment for leiomyosarcoma, to remove as much of the tumor as possible and eradicate any remaining malignant cells with radiation. Chemotherapy can be used in cases of metastasis or if the disease recurs after initial treatment [9]. Chemotherapeutic agents usually used in SST include doxorubicin and ifosfamide, gemcitabine and Taxotere (docetaxel), dacarbazine, and ecteinascidin. Based on a meta-analysis, adjuvant doxorubicin-based chemotherapy improved time to local and distant recurrence rates, as well as overall recurrence-free survival [17,18]. The median survival time for leiomyosarcoma of the thigh alone, as well as with lung metastasis, varies widely and is dependent on several factors, including tumor size, tumor location, the extent of the metastasis, patient age, comorbidities, and the effectiveness of the treatment. However, according to some studies, for patients with localized leiomyosarcoma of the thigh, the median survival time is reported to be between 50 to 60 months. In contrast, the median survival time for patients with metastatic leiomyosarcoma to the lung is 12 to 15 months [12]. 

## Conclusions

Leiomyosarcoma is an aggressive tumor that arises from various locations. Only a few case reports are available on leiomyosarcoma of the thigh, and limited published data on patients with leiomyosarcoma of the SST. The overall survival rate of leiomyosarcoma of SST is low due to the poor prognosis. If imaging studies suggest a sarcoma, it is imperative not to biopsy prior to surgical removal, as disrupting SST leiomyosarcomas increases the risk of metastasis. The rarity of these tumors makes it challenging to create definitive treatment protocols. However, the accepted treatment for leiomyosarcoma is surgery with wide-margin removal. As for leiomyosarcomas in the extremity, chemotherapy and radiation can be used depending on further evaluation and metastasis risk assessment. We hope our case might be a valuable addition to the medical literature to help understand the behavior of this rare tumor and influence future treatment guidelines.
